# The Effect of Female Personnel on the Voluntary Disclosure of Carbon Emissions Information

**DOI:** 10.3390/ijerph192013247

**Published:** 2022-10-14

**Authors:** Eunsoo Kim

**Affiliations:** Department of Global Business Administration, Sangmyung University, Seoul 03016, Korea; eunsoo1134@gmail.com

**Keywords:** female personnel, ESG investments, voluntary disclosure, carbon emissions information

## Abstract

This paper examines the effect of female personnel (female executives and female employees) on corporate voluntary disclosure policy on carbon emission information. The study is motivated from the recent fact that the laws and systems related to female workers are rapidly changing as the social and economic status of women in South Korea has recently improved. In a sample of 9406 firm-year observations over the period from 2014 to 2020, the higher the proportion of female executives, the higher the frequency of voluntary disclosure on carbon emission information. These results are the same even when the female workforce was measured as the proportion of general female employees. Therefore, it can be said that the existence of female personnel at the management and practice level plays a significant role in improving voluntary disclosure quality. By considering the impact of gender manpower composition on enhancing corporate transparency, it provides evidence that market participants can have a positive view on the quality of information environment provided by companies with a high proportion of female personnel.

## 1. Introduction

Paying attention to the rapid change in laws and systems related to female human resources due to the recent increase in their social and economic status, this study aims to examine the impact of female professional manpower on the voluntary disclosure of greenhouse gas emission information. As female social and economic power grows around the world, the trend of the ‘sheconomy’, in which women act as important players in economic decision-making is spreading. ‘Sheconomy’ is the first term used by *The Times* in 2010 in the United States, focusing on the fact that female consumers account for more than 80% of the total purchase decisions. This is a combination of ‘she’, a pronoun for women, and ‘economy’, which means ‘female economy’, in which women are the main economic players. The ‘sheconomy’ craze, which began in the United States, is appearing simultaneously in major countries as the female economic population increases amid changes in the global industrial structure. In particular, female power has been emphasized not only in terms of consumption but also in perspective of supply entities. As more and more women occupy major positions in the top management team of companies recognized as having glass ceiling, interest in women’s position is being re-examined.

In the case of South Korea, due to a notion of preferring a son to a daughter in the past, the proportion of women among the total population was low and female economic activity was very low. In South Korea, where the remnants of Confucian culture (predominance of man over woman) of the Joseon Dynasty remained, and the heavy and chemical industries such as automobiles, shipbuilding, and chemicals had compressed growth in the 70s and 80s, there were differences in perception of gender responsibility and role as economic agents. However, due to the recent trend of changing perception of gender equality, the number of female populations has increased, and female economic activities are increasing as the level of education has improved significantly. In addition, many companies have been trying to improve the awareness of female workers in the organization and establish a gender-equal corporate culture. Lotte Group, for example, announced its ‘Diversity Charter’ in 2013 aimed at respecting diversity and eliminating discrimination among its members, selecting more than 40 percent of new employees as women, and holding a women-related forum in the company. Hanssem also wanted to establish a ‘corporate culture room’ in the company, and HP in Korea sets a target ratio of female employees and monitors them from time to time and organizes a ‘WAVE (Woman Adding Value with Enthusiasm)’ to consider and solve gender equality issues. In terms of national policy, the introduction of a ‘female executive quota system’ (in effect from July 2020) was decided in early 2020 through the revision of the Capital Market and Financial Investment Business Act, which mandates the appointment of female registered executives for listed companies with total assets of 2 trillion won. As such, interest in the role of female manpower is increasing in line with the recent trend of ‘sheconomy’ in advanced countries in South Korean society.

On the other hand, research has been actively conducted to focus on corporate governance as a major factor affecting disclosure quality as voluntary disclosure is evaluated to have a positive function of resolving the information asymmetry between managements and investors and inducing efficient allocation of scarce resources. In general, companies with good corporate governance are considered to have high disclosure quality because they are interested in transparency in information environments and carefully consider reputation and legal issues [1,2,3,4]. In a study of domestic companies, it is reported that the level of corporate governance measured by the characteristics of the board of directors (independence, expertise, activity), the introduction of an audit committee, and foreign investors’ ownership has a significant effect on the disclosure quality [5,6,7].

However, there are no studies that pay attention to the composition of manpower, particularly gender diversity, in relation to a corporate disclosure policy in South Korea. Therefore, this study focuses on the recent changes in the legal and social environment of South Korean society and examines the impact of female manpower on the quality of disclosure. There is an opinion that the proportion of female workers can have a positive effect on disclosure policies and corporate value in the long run by enabling 21st century emotional management that meets corporate transparency, fairness, diversity, and customer needs [8]. Specifically, the effect of the increase in the proportion of female workers in the organization is as follows. First, from the perspective of viewing the gender of women as a kind of resource, considering that most of the existing workers in the company are men, having female workers can be a driving force to act as a kind of competitive advantage. As mentioned earlier, the fact that women are easier than men to grasp the needs of women, which are major consumers, may be a competitive factor for female workers. Second, gender diversity, which increases by hiring women within existing male-centered corporate organizations, provides different characteristics and perspectives, allowing them to establish management strategies or present more diverse perspectives and solutions when faced with certain problems. In this way, the gender diversity that appears by hiring women is likely to affect corporate performance and long-term growth opportunities. In summary, women act as a new factor in the existing male worker-centered corporate organization through various channels.

As a result of analyzing listed companies in South Korea, it was found that the greater the proportion of women among board members, the greater the tendency to voluntarily disclose carbon emission information. In addition, the larger the proportion of female workers among all personnel, the more voluntary disclosure of carbon emission information was being made. As a result of further analysis, the effect of female personnel on the disclosure policy seems to be more promoted by particularly encouraging investment in environmental aspects among ESG investments. These results were reported qualitatively the same in the robustness analysis that controlled the potential endogeneity of female executive variables. Therefore, it can be said that the presence of female manpower (executives and personnel) at the corporate management and practice contributes to improving the quality of disclosure.

The contributions of this study are as follows. This study expands the scope of research that can explain the factors affecting voluntary disclosure policies in terms of corporate governance by examining the relationship between female manpower and disclosure quality. In addition, by examining the impact of gender workforce composition to enhance corporate transparency through voluntary disclosure, it provides a basis for having a positive view on carbon emission information provided by companies with a high proportion of female workforce when making investment decisions on those firms. Furthermore, it provides policy implications for the necessity of gender equality manpower policy in relation to recent institutional changes in South Korea, such as the introduction of the ‘female executive quota system’.

The composition of this study will proceed in the following order. In the second chapter, previous studies are reviewed, and hypotheses are established. The third chapter designs a research model and describes the process of data collection. The next chapter describes the results of empirical analysis, and the last chapter summarizes the research contents and presents conclusions.

## 2. Backgrounds and Hypotheses

Corporate disclosure is a system in which a company informs the market of information necessary for decision-making by external stakeholders. Companies have used financial policies such as dividends, stock split, and treasury stock acquisition as major signaling means of delivering internal information to the capital market, and the role of disclosure policies is expanding as a means of reducing information asymmetry between managers and external stakeholders. Disclosure is evaluated as helping to solve problems such as reverse selection, undervaluation of corporate value, and high financing cost by reducing information asymmetry between companies and investors, and ultimately inducing efficient allocation of scarce resources.

Verrecchia (1982) and Diamond (1985) argued that if a company provides a lot of public information, the incentive to search for private information is reduced, and the information asymmetry between investors is resolved [9,10]. Merton (1987) explained that disclosure improves corporate transparency through the investment recognition hypothesis, thereby reducing information risk between investors with different level of information [11]. Healy et al. (1999) reported that the number of stock price returns, stock liquidity, institutional investors, and financial analysts increases in companies with a higher level of disclosure quality [12]. Lang and Lundholm (1996) reported that the higher the level of disclosure, the higher the number of financial analysts, and the smaller the earnings forecast variance and error of financial analysts, explaining that disclosure can lower investors’ predictive risk in the capital market and reduce information asymmetry [13].

Disclosures are divided into regular disclosures that regularly disclose business reports, etc., and irregular disclosures that frequently disclose major management matters. The disclosure used in this study corresponds to an irregular disclosure and include in a voluntary disclosure that varies depending on the manager’s choice. Specifically, voluntary disclosure is called so because managers voluntarily choose whether to disclose information on corporate carbon emissions, and there is no special penalty for not disclosing due to the nature of voluntary disclosure. Recently, research on the disclosure of carbon emission information by companies has been actively conducted, but only a small number of studies have been conducted in South Korea for the reason of securing data [14,15,16,17,18]. Briefly introducing domestic studies conducted with carbon emission information data surveyed by CDP institutions such as this paper, there are studies showing that the higher the carbon emission information, the higher the corporate value, and the higher the share of foreign investors, the higher the tendency to disclose carbon emission information.

The Carbon Disclosure Project (CDP) was founded in December 2000 under the auspices of 35 European financial institutions ($4 trillion). Since then, the number of member institutions has steadily increased, reaching 822 CDP-signed financial institutions (operating assets: $95 trillion) as of 2015, and more than 5500 companies around the world provide their climate change information to investors through CDP. As of 2015, 31 financial institutions in South Korea have joined as members and are collecting related information from 250 listed companies. On behalf of financial institutions around the world, the CDP requires and collects accurate information on major greenhouse gas emissions, including carbon dioxide, a major cause of climate change, and corporate short- and long-term management strategies on related topics. It also provides information for investors to avoid risks by identifying the level of climate change risk of a particular company in advance.

Companies subject to the carbon emission information survey voluntarily fill out responses to the CDP’s questionnaire every year, and its contents are evaluated by the method jointly developed by the CDP and PwC. For domestic companies, the CDP Korea Committee will partially revise the method to suit the domestic situation to evaluate the response content, and the evaluation results will be released through the CDP official website (http://cdp.net, (accessed on 1 January 2021)) and the CDP final report. Corporate response data are analyzed and evaluated in two aspects: performance and disclosure. Performance scores assess the level of climate change activities reported by companies, such as climate change mitigation, adaptation, and transparency, and open scores assess whether the company responded appropriately according to the completeness, quality, and format of CDP questions. Companies with high performance or disclosure scores are also included in the A List (performance band A) or the Climate Disclosure Leadership Index (CDLI). The CDP results report discloses the corporate response to the CDP survey, performance bands and public scores, scope1 emissions (direct emissions), scope2 emissions (indirect emissions), number of reports emitted to scope3 (other indirect emissions not included in scope2), verification of scope1, 2, 3, and reduction targets. Among them, the open score is 75 or higher, and all open scores and performance bands of companies that choose “Public” are disclosed when submitting CDP, but if the score obtained is below the disclosure standard (75 points), the open score and performance band are not disclosed. In this way, providing and disclosing carbon emission information to the CDP is an option, not an obligation, but the carbon emission information disclosed through the CDP is evaluated to be very reliable on the following grounds [19]. The market can evaluate the reliability of the disclosed carbon emission information by comparing the carbon emission information of a company with other firms in the same industry. The CDP conducts a survey of major companies around the world, and in some countries, such as the EU, emissions are legally regulated. In this case, since the accuracy of carbon emission information is secured and these companies are subject to comparison with other firms, companies that voluntarily disclose information can secure reliability by using it as a reference standard.

While disclosure is evaluated to have a positive function to resolve information asymmetry between managements and investors and induce efficient allocation of limited resources, research has been actively conducted focusing on corporate governance as a major factor affecting disclosure quality. In general, companies with good governance structure are reported to have high disclosure quality because they are relatively interested in information transparency and carefully consider corporate reputation and legal issues. Chen and Jaggi (2000), Engand Mak (2003), and Ajinkya et al., (2005) reported that the lower the number of outside directors, the lower the level of disclosure [1,2,3]. Hope and Thomas (2008) argued that the level of disclosure is low in companies that do not have a device to supervise and check management [4]. Chen et al. (2008) report that family firms voluntarily disclose earnings forecasts or have conference calls compared to non-family companies [20]. However, among these previous studies, few studies have analyzed corporate workforce composition, especially gender diversity, in terms of governance. Therefore, this study focuses on the recent changes in the legal and social environment of South Korean society and examines the impact of female manpower on the voluntary disclosure policy on carbon emissions.

Female workers themselves have a positive effect on companies as human resources, but they also have a positive effect on companies through synergy between members as they enhance diversity within the organization. In addition, the presence of female workers in companies is likely to affect corporate performance and long-term growth through external factors as well as unique differences or synergy within the organization, supported by various policies for female employment. For example, if female workers properly grasp the consumption patterns and trends of the same female consumer and reflect them in the corporate business activities, the company’s market share with female customers will increase. Additionally, if it is leveled by the presence of female workers in the vertical decision-making structure centered on men in the company, more diverse ideas will be created and an important factor that can induce corporate innovation. Furthermore, the image of a ‘gender equality company’ obtained by actively hiring women is an important factor in securing competent female workers in the labor market. In addition, the higher the proportion of female workers, the higher the possibility of policy support or the lower regulatory violations, which is difficult to express in certain figures, are factors that can ultimately have a positive effect on companies.

According to the upper echelon theory of Hambrick and Mason (1984), the strategic choice and performance of the organization are influenced by the background characteristics of managers [21]. In particular, looking at gender studies, women have fundamentally different characteristics (ex. value, perception, preference, attitude) from men [22]. There is also an opinion that gender differences are small among executives located at the top managements due to socialization in the field, but gender differences are often maintained due to human general characteristics [23,24]. First, women are evaluated as risk-averse and have conservative tendencies over men [25,26,27]. Sundenand Surette (1998) argued that women choose products with lower risk factors than men when choosing pension funds [25]. Olsen and Cox (2001) reported that female investment experts focus more on reducing risks such as the possibility of loss and uncertainty in constructing a portfolio than male investment experts [26]. Women are also reported to have higher moral and ethical standards than men [28,29]. Ford and Richardson (1994) looked at thirteen previous studies on ethical decision-making by gender, and found evidence that women make ethical decisions more than men in eight studies [28]. Bernardi and Arnold (1997) confirmed that female moral standards were higher than men in a survey on necessary decision-making in social dilemma situations [29].

As women’s entry into the upper class has increased rapidly in recent years, research on the effect of female executives on major decision-making and performance of companies has been actively conducted. First, looking at a study on the effect of female executives on corporate management decision-making, Barber and Odean (2001) argued that female directors have a lower tendency to be overconfident than men, so relatively high standards are applied in monitoring managers [30]. Heminway (2007) reported that female executives are less likely to arbitrarily adjust corporate financial disclosures because they have higher reliability standards than men [31]. Huang and Kisgen (2008) found that female CFOs make more careful decisions when making management decisions such as corporate acquisition or debt issuance than male CFOs [32]. Adams and Ferreira (2009) reported that the more female executives attend the board of directors meeting than male directors, and the higher the ratio of female directors within the board of directors, the stronger the monitoring function for controlling shareholders and managements [33]. Barua et al. (2010) suggested that companies with female CFOs have high quality of accruals due to characteristics such as risk-averse attitudes and regulatory compliance [34]. Gul et al. (2011) argued that the more female executives in the board of directors, the higher the quality of profits and the greater the usefulness of accounting information [35]. Srinidhi et al. (2011) presented that the earnings managements through discretionary accruals is reduced when there are female executives in a company and interpreted that the quality of earnings has improved [36]. Francis et al., (2015) reported that when the gender of the CFO changes from male to female conservative accounting increases as the tendency to avoid litigation risk, default risk, and defamation risk increases [37].

Taken together, women can improve the usefulness of accounting information while faithfully performing the role of monitoring and checking as board members based on biological characteristics such as conservatism and morality [31,34,35,36,37]. Female executives are also evaluated as helping to form a transparent and efficient organizational culture and ultimately enhancing corporate reputation and value by actively presenting various opinions from an independent perspective based on the characteristics of independence, activity, and gender diversity [33,38]. In particular, female executives are more likely to induce companies to faithfully perform their obligations from a conservative perspective because they tend to avoid various risks that may occur compared to men. Considering previous studies that female executives have a positive effect on corporate governance, companies with female executives are expected to more actively implement voluntary disclosure policies on carbon emission information, so the following Hypothesis 1 is established.

**Hypothesis** **1.** *Female representation on boards is positively associated with voluntary disclosure of carbon emission information.*

At the field of practice, female employees can also play a positive role in enhancing corporate value as they present various perspectives and contribute to forming a flexible organizational culture when making corporate management decisions. This can be explained by diversity theory. Diversity theory explains the relationship between various categories such as gender, age, race, knowledge, education, and values of members of a company and corporate management. Diversity theory focuses not on the strategic characteristics of women themselves, but on the positive effects that appear when women are put into existing organizations to enhance organizational diversity. Most previous studies reported that diversity of organizational members in companies improves management performance, and they also argued that by hiring female personnel to utilize the delicate sensibility of female manpower in the 21st century, new perspectives of women can be applied to management to accelerate corporate innovation [39].

Second, it can be explained by institutional theory. This can be seen as the perspective of companies hiring women due to social factors such as forced employment by state policy, jumping on the social trend of increasing female employment rate, competition to attract excellent female manpower, and increased reputation obtained by hiring women. Scott (2008) defined institutional factors by largely dividing them into regulatory, normative, and cognitive systems, and stated that the pressure from each system changes the organizational structure of a company [40]. They also reported that properly complying with the system applied to companies in various forms increases organizational performance because social resources and support can be allocated.

Overall, female employees are valued as differentiated human resources from men and have relatively higher moral and ethical standards than men, so the presence of female employees in companies is expected to have a positive effect on the fulfillment of disclosing activities [28,29]. Therefore, this study establishes the following Hypothesis 2.

**Hypothesis** **2.** *Female personnel is positively associated with voluntary disclosure of carbon emission information.*

## 3. Research Design and Sample Description

### 3.1. Research Design

The following regression analysis model was used to verify the relationship between female executives and voluntary disclosures on carbon emissions.

(1) $$\begin{array}{l} VDCE_{t}=\alpha_{0}+\beta_{1}FB_{t}+\beta_{2}SIZE_{t}+\beta_{3}LEV_{t}+\beta_{4}GROW_{t}+\beta_{5}ROA_{t}+\beta_{6}AQ_{t}+\\ \beta_{7}MO_{1}+\beta_{8}FO_{t}+\sum IND+\sum YR+\varepsilon_{t} \end{array}$$

where *VDCE* = 1 if firms report carbon emission information voluntarily, and 0 otherwise; *FB* = the proportion of female executives in board of directors; *SIZE* = the total asset of firm, linearized with natural log; *LEV* = the total liability of firm, standardized by the total asset; *GROW* = market value of equity/book value of equity; *ROA* = net income/total asset; *AQ* = earnings quality; *MO* = majority shareholders’ ownership; *FO* = foreign investors’ ownership.

Equation (1) includes year fixed effect dummies and industry fixed effect dummies to incorporate for variations within companies in the similar year-industry observations. *VDCE* is measured as a dummy variable and the value of *VDCE* is 1 if companies report carbon emission information voluntarily. The test variable is *FB* and it is measured by the proportion of female executives in board of directors. Significantly positive/negative coefficient on *FB* in Equation (1) indicates that the Hypothesis 1 is supported.

Second, Hypothesis 2 predicts whether female personnel impact voluntary disclosure of carbon emission information.

(2) $$\begin{array}{l} VDCE_{t}=\alpha_{0}+\beta_{1}FP_{t}+\beta_{2}SIZE_{t}+\beta_{3}LEV_{t}+\beta_{4}GROW_{t}+\beta_{5}ROA_{t}+\beta_{6}AQ_{t}+\\ \beta_{7}MO_{1}+\beta_{8}FO_{t}+\sum IND+\sum YR+\varepsilon_{t} \end{array}$$

where *FP* = female personnel/total personnel.

In Equation (2), dependent variable is *VDCE* and test variable is *FP*. A significantly positive/negative coefficient on *FP* implies that the Hypothesis 2 is supported.

I control for certain characteristics that can influence corporate voluntary disclosure of carbon emission information. These are firm size (*SIZE*), debt ratio (*LEV*), market-to-book ratio (*GROW*), return on asset (*ROA*), accrual quality (*AQ*), majority shareholders’ ownership (*MO*), foreign ownership (*FO*). Among the control variable, *AQ* is measured by the research model in Kothari et al. (2005), and the specific model to measure *AQ* is presented below in Equation (3) [41]:

(3) $$\frac{TA_{t}}{A_{t1}}=\alpha_{0}+\beta_{1}\frac{1}{A_{t1}}+\beta_{2}\frac{\Delta S_{t}\Delta AR_{t}}{A_{t1}}+\beta_{3}\frac{PPE_{t}}{A_{t1}}+\beta_{3}ROA_{t}+\varepsilon_{t}$$

where *TA* = Net income − cash flow from operations; *S* = Sales revenue; *AR* = Accounts receivables; *PPE* = Plant, property, and equipment; *ROA* = Net income/total assets; *A* = Total assets.

To measure accrual quality, a cross-sectional model of discretionary accruals is employed. Specifically, it is estimated by all the industries incorporated in the study which are classified by its two-digit industry codes. Final data includes only firms with minimum 20 firm-year observations to guarantee efficient sample for calculation. This study used residuals to measure *AQ* which are calculating from Equation (3). For the easier interpretation, *AQ* is acquired by multiplying the negative one. The detailed definition of all the control variables is shown below each equation.

### 3.2. Sample

The sample period is from 2014 to 2020. Sample firms are collected from the Korea Stock Market, including KOSPI and KOSDAQ specifically, but firms in the finance and insurance industry are not included. Table 1 describes the sample selection procedures and the composition of samples used for empirical analyses. The number of non-financial firms with a December year-end 12,951 from 2014 to 2020. I exclude firms with incomplete financial statement variables in the database. Then, firm-year observations without female executives or female personnel variables in the database are excluded. After that, I winsorize the top and bottom 1% of the main variables to reduce the outlier effects. Finally, through this process, this study gets 9406 firm-year observations for the research.

## 4. Findings

### 4.1. Descriptive Statistics

Descriptive statistics for main variables are presented in Table 2. The mean value of VDCE is 0.0280, indicating that the average voluntary disclosure frequency is 2.80% over 6 years. The mean of FB implies that sample firms show 23.25% of female executives on average. Finally, the average value of FP is 3.7635 and its median is 3.5835.

Table 3 presents Pearson correlation coefficients for the main variables used in this paper. I find that voluntary disclosure of carbon emission (*VDCE*) has significant positive correlation with female board of directors (*FB*), consistent with Hypothesis 1. Additionally, positive association is discovered on FP (female personnel) with the 1% significance. These results indicate that firms with higher proportion of female executives and female personnel are more likely to voluntarily disclose carbon emission information, although the factors affecting *VDCE* has not been properly controlled.

### 4.2. Empirical Findings

#### 4.2.1. Regression Results of H1

Table 4 represents the regression results of testing Hypothesis 1 using Equation (1). Specifically, I first test whether female board of directors induce voluntary disclosure of carbon emission information. Table 4 shows that the coefficient of the test variable (*FB*) is significantly positive with 1% significance level, which offers support for Hypothesis 1. Moreover, significant relationship between control variables (*SIZE, LEV, GROW, ROA, AQ, MO, FO*) and voluntary disclosure of carbon emission information are also represented. Some of variables such as *SIZE, GROW, AQ*, and *FO* show a significant positive relationship to *VDCE*, and the others (*LEV, ROA, MO*) illustrate a negative relationship with 1% significance.

#### 4.2.2. Discussion of H1

McKinsey (2001) suggested that the higher the ratio of female executives, the higher the welfare of shareholders, and also argued that the active use of female manpower as executives helps female executives to implement environmentally friendly policies and ultimately induce voluntary disclosure of carbon emission information, resulting in improved corporate value from a long-term perspective [42]. In other words, when female manpower is employed full-time and leadership is exercised to members as executives, diversity within the organization is secured through female unique meticulousness, empathy, and communication ability, which can induce voluntary disclosure to resolve information asymmetry.

Overall, it is judged that female executives in top management teams are giving a positive influence to companies in the long-term by improving the disclosure environment with a new perspective. In order to secure long-term growth with improved disclosure policy, companies need to increase the proportion of female executives in the organization. Additionally, at the same time, there is a need to improve awareness of female executives in the organization and to support institutional mechanisms so that female human resource factors can be sufficiently expressed within the company.

#### 4.2.3. Regression Results of H2

Table 5 shows the results of the regression analysis using Equation (2). Hypothesis 2 examines the influence of female personnel on voluntary disclosure of carbon emissions information. As shown in Table 5, the coefficient of *FP* is significantly positive at the 1% level. This is consistent with the prediction that female manpower affects voluntary disclosure policy. Significant relationship is also displayed between the control variables and voluntary disclosure of carbon emission information. Some of the control variables (*SIZE, GROW, AQ, FO*) have a significant positive association with *VDCE*, and others (*LEV, ROA, MO*) show a negative relationship.

#### 4.2.4. Discussion of H2

Female workers are innovative as a resource value, relationship-oriented, and have an interdependent property of learning in cooperation with others [43]. In addition, there is a strong distinction that it can maximize demand identification or satisfaction with female customers who play a major consumer role in the family [39]. Overall, female workers can present new perspectives and problem-solving solutions rather than existing methods. As a result, female personnel can form a social friendly image of a company that aims for gender equality and contributes to build an environmentally friendly image by reducing information asymmetry through voluntary disclosure of carbon emission information [44].

### 4.3. The Effect of ESG Investments

Disclosures generally play a role in promoting efficient allocation of resources by lowering the information asymmetry that occurs between managers and investors [12]. By voluntarily disclosing reliable carbon emissions information, companies communicate to the market about future possible costs of carbon emissions. This reduces uncertainty about future cash flows, thereby reducing the cost of capital and increasing corporate value [45,46,47,48]. Although disclosure of carbon emission information is at the discretion of managements, as the number of companies disclosing that information within the same industry increases and the demand for carbon emission information from stakeholders rises, it became a significant disclosure threat to individual companies [19]. Through the main regression results, this study reported the effect of female manpower on voluntary disclosure on carbon emissions, which plays a role in increasing corporate value from a long-term perspective. However, it has not been reported that which aspects of the corporate activities are affected by female manpower which leads to voluntary disclosure of environmental issues. For the long-term survival and sustainable growth of a company, ESG activities emphasizing not only financial factors such as economic activities but also non-financial factors such as eco-friendly management, social responsibility, and governance improvement are needed [49]. Since the voluntary disclosure policy, which is the focus of this study, has some stronger non-financial aspects than financial activities, this study conducted an additional analysis to examine which aspects of ESG investment activities affect and ultimately induce voluntary disclosure.

Most ESG-related studies in South Korea were conducted based on the ESG evaluation score of the Korea Corporate Governance Service (KCGS). The KCGS has evaluated the level of corporate governance (G) since 2003, and has evaluated the level of ESG by adding environmental (E) and social responsibility (S) items since 2011. Environmental items are evaluated as environmental strategies, environmental organizations, environmental management, environmental performance, and stakeholder response, and social responsibility (S) items are evaluated as workers, partners and competitors, consumers, and local communities. The corporate governance (G) items are evaluated as shareholder rights protection, board of directors, audit organizations, and disclosure sectors, and the total score for each item is 300. The KCGS announces seven grades of S, A+, A, B+, B, C, and D based on the scores of each item. In this study, the ESG score of the KCGS was used as a measure of the ESG activity level (ESG) of a company.

As a result of the analysis in Table 6, it was found that female manpower had the greatest influence on ‘E’ item among ESG investment activities, which leads to voluntary disclosure policies. After the environmental aspects, it appeared in the order of corporate governance (G) and social responsibility activities (S). These results can be explained that female manpower has a great influence on the environmental aspects of non-financial activities of companies, thereby promoting voluntary disclosure policies on carbon emissions.

### 4.4. Robustness Regression

This study conducts a robustness analyses using fixed-effect model to control for the effect of outlier bias. These type of regression techniques can reduce the problem of omitted variable induced by hidden heterogeneity which is constant over time. This type of heterogeneity problem can be managed from subtracting the group-level mean over time.

Table 7 shows the results through fixed-effect model and the coefficients of FB and FP are positively significant, respectively, which all support Hypotheses 1 and 2. Collectively, as can be discovered in Table 7, the results of this study maintain consistent findings with the main model even with the robustness test.

## 5. Conclusions

This study focused on the rapid change in laws and systems related to female manpower due to the recent increase in the social and economic status of women in South Korea, and examined the impact of female manpower on corporate voluntary disclosure policies on carbon emission information. In the past, South Korea had a low proportion of women and very little economic activity among the total population due to the preference for sons, but economic activity has been increasing as women’s educational level has improved significantly due to recent changes in perceptions of gender equality. In terms of national policy, interest in the role of female manpower is also increasing, such as introducing the ‘gender equality employment quota system’, ‘maternity leave’, and ‘women executive quota system.’ With attention to the increasing economic status of women, the paper investigates the impact of female manpower in terms of gender diversity on the disclosure policy that play a role in resolving information asymmetry in the capital market and inducing efficient allocation of limited resources.

As a result of an empirical analysis of domestic listed companies, it was found that the higher the proportion of female executives and female personnel, the higher the frequency of voluntary disclosure of carbon emission information. In particular, it was found that women had the greatest influence on environmental investment among corporate ESG investments, inducing them to actively promote voluntary disclosure policies on carbon emissions. In addition, qualitatively the same results were reported in the robustness analysis that controlled the potential endogeneity of female executive variables. Therefore, through this study, it can be concluded that at the management and practical level of companies, female manpower contributes to improving the quality of voluntary disclosure of carbon emission information.

However, since this study only analyzed South Korean listed companies requested by CDP institution, the number of samples was not relatively large and specified by industry, so there is a limitation in that the relationship between female workers and carbon emission disclosure of companies was not verified specifically. Furthermore, the higher the proportion of female CEOs and female executives, the higher the proportion of female workers, and if the corporate value is high as a female CEO, the impact of the ratio on corporate disclosure policies may simply be determined endogenously. Another caveat is the following. Since the finding in this study focus only on companies in a single country, the results of an overall positive relationship between female executives (personnel) and disclosure of carbon emissions may not generalize to companies in other countries that are subject to more severe discrepancy in gender equality than South Korea. Thus, future research can be conducted whether the findings in this study extend to companies operating in other countries that are known for severe gender inequality in work.

Despite the above weaknesses, this paper can contribute to the prior research in the following ways. First, this study expands the scope of research that can explain the factors affecting the quality disclosure in terms of corporate governance by examining the relationship between female manpower and voluntary disclosure quality on environmental issues of companies. By examining the impact of gender manpower composition to enhance corporate transparency, it provides a basis for having a positive view on information provided by companies with a high proportion of female manpower when making investment decisions by market participants.

Second, it provides policy implications for the necessity of gender equality manpower policy in relation to recent institutional changes in South Korea, such as the introduction of the ‘female executive quota system’. According to the Economist in 2014, it was revealed that South Korean women are being treated as the worst gender inequality among OECD countries in the workplace as South Korea ranks at the bottom of the Glass Ceiling Index among OECD countries. In addition, statistics reported by the OECD in 2015 showed that the salary gap between South Korean women and men was the largest among OECD member countries, indicating that South Korean female workers had very little decision-making power in the workplace. Since the first female president was born in South Korea in 2013, the absence of female executives at the top of the company has led to strong pressure to increase the proportion of female executives and employees. Accordingly, in January of the same year, the gender quota system was legislated to increase the ratio of female board members to 15% for three years in public companies, and it was institutionalized to expand to 30% within five years [50]. In spite of the legislative and social moves towards gender diversity, little empirical evidence has found on the potential benefits related to female representation in executives and personnel. Moreover, this study contributes to a better understanding of when female manpower plays a role in credible environmental disclosure.

## Figures and Tables

**Table 1:** The Sample Selection Procedure.

**Panel A: The Data Selection Process**	
Total listed non-financial firms with December year-end	12,951
(-):	
Firms without necessary data for control variables	1309
Firms without female executives or female personnel	2236
Final observation	9406
**Panel B: Industry Distribution**	
Industry	Number (%) of Firms
Foods/Tobacco	345
Textiles/Shoes/Bags	217
Woods/Pulp/Paper/Prints	185
Chemicals/Plastics/Rubber	1517
Nonmetals	174
Primary Metals/Metalworking Processes	648
Machinery/Computer/Vehicle	3241
Construction	239
Wholesale/Retail	759
Services	2081
Total	9406

**Table 2:** Descriptive Statistics.

Variables	Mean	STD	Q1	Median	Q3
*VDCE*	0.0280	0.1651	0.0000	0.0000	0.0000
*FB*	0.2325	0.4394	0.0000	0.0000	0.6931
*FP*	3.7635	1.4398	2.7725	3.5835	4.5432

Notes: Variable definition: *VDCE* = 1 if firms report carbon emission information voluntarily, and 0 otherwise; *FB* = the proportion of female executives in board of directors; *FP* = female personnel/total personnel.

**Table 3:** Pearson Correlation.

	(1)	(2)	(3)
(1) *VDCE*	1.0000	0.0305	0.3298
		<0.0001	<0.0001
(2) *FB*		1.0000	0.3431
			<0.0001
(3) *FP*			1.0000
		

**Table 4:** The regression result of H1.

Variables	Coeff.	t-Stat.
*Intercept*	−1.3245	−35.08 ***
*FB*	0.0127	13.44 ***
*SIZE*	0.0520	35.34 ***
*LEV*	−0.0046	−2.80 ***
*GROW*	0.0039	4.31 ***
*ROA*	−0.1102	−6.41 ***
*AQ*	0.0488	2.65 ***
*MO*	−0.0774	−7.99 ***
*FO*	0.1450	8.33 ***
*IND Dummy*	Included	
*YEAR Dummy*	Included	
F-value	140.31 ***	
Adj. R^2^	0.245	
Observations	9406	

(1) *** indicates significance at the 1% levels. (2) *FB* = the proportion of female executives in board of directors; *SIZE* = the total asset of firm, linearized with natural log; *LEV* = the total liability of firm, standardized by the total asset; *GROW* = market value of equity/book value of equity; *ROA* = net income/total asset; *AQ* = earnings quality; *MO* = majority shareholders’ ownership; *FO* = foreign investors’ ownership.

**Table 5:** The regression result of H2.

Variables	Coeff.	t-Stat.
*Intercept*	−1.2881	−31.39 ***
*FP*	0.0099	7.14 ***
*SIZE*	0.0495	29.41 ***
*LEV*	−0.0065	−3.96 ***
*GROW*	0.0040	4.40 ***
*ROA*	−0.1230	−7.08 ***
*AQ*	0.0587	3.17 ***
*MO*	−0.0828	−8.49 ***
*FO*	0.1553	8.85 ***
*IND Dummy*	Included	
*YEAR Dummy*	Included	
F-value	132.63 ***	
Adj. R^2^	0.235	
Observations	9406	

(1) *** indicates significance at the 1% levels. (2) *FP* = female personnel/total personnel. (3) See Table 4 for definitions of other variables.

**Table 6:** The effect of ESG investments.

**Panel A. E Investments**				
	**Female Board (H1)**		**Female Personnel (H2)**	
**Variables**	**Coeff.**	**t-Stat.**	**Coeff.**	**t-Stat.**
*FB/FP*	0.0320	4.15 ***	0.0081	2.87 ***
*Control Variables*	Included		Included	
F-value	79.22 ***		75.12 ***	
Adj. R^2^	0.304		0.296	
Observations	4283		4219	
**Panel B. S Investments**				
	**Female Board (H1)**		**Female Personnel (H2)**	
**Variables**	**Coeff.**	**t-Stat.**	**Coeff.**	**t-Stat.**
*FB/FP*	0.0283	3.59 ***	0.0018	0.65
*Control Variables*	Included		Included	
F-value	79.79 ***		76.93 ***	
Adj. R^2^	0.306		0.301	
Observations	4283		4219	
**Panel C. G Investments**				
	**Female Board (H1)**		**Female Personnel (H2)**	
**Variables**	**Coeff.**	**t-Stat.**	**Coeff.**	**t-Stat.**
*FB/FP*	0.0284	3.54 ***	0.0058	2.21 **
*Control Variables*	Included		Included	
F-value	83.27 ***		79.50 ***	
Adj. R^2^	0.285		0.280	
Observations	4283		4219	

(1) **, and *** indicate significance at the 5%, and 1% levels, respectively. (2) See Table 4 for definitions of other variables.

**Table 7:** Robustness Regression.

**Panel A. Hypothesis 1**		
**Variables**	**Coeff.**	**t-Stat.**
*FB*	0.0433	11.85 ***
*Control Variables*	Included	
F-value	147.53 ***	
Adj. R^2^	0.265	
Observations	9406	
**Panel B. Hypothesis 2**		
**Variables**	**Coeff.**	**t-Stat.**
*FP*	0.0099	7.17 ***
*Control Variables*	Included	
F-value	142.30 ***	
Adj. R^2^	0.258	
Observations	9406	

(1) *** indicates significance at the 1% levels. (2) See Table 4 and Table 5 for definitions of other variables.
